# Developmental Paths of Pointing for Various Motives in Infants with and without Language Delay

**DOI:** 10.3390/ijerph19094982

**Published:** 2022-04-20

**Authors:** Katharina J. Rohlfing, Carina Lüke, Ulf Liszkowski, Ute Ritterfeld, Angela Grimminger

**Affiliations:** 1 Faculty of Arts and Humanities, Paderborn University, 33098 Paderborn, Germany; angela.grimminger@upb.de; 2 Fakultät für Humanwissenschaften, University of Würzburg, 97074 Würzburg, Germany; carina.lueke@uni-wuerzburg.de; 3 Institut für Psychologie, University of Hamburg, 20146 Hamburg, Germany; ulf.liszkowski@uni-hamburg.de; 4 Fakultät für Rehabilitationswissenschaften, TU Dortmund University, 44227 Dortmund, Germany; ute.ritterfeld@tu-dortmund.de

**Keywords:** pointing gestures, pointing motives, developmental paths

## Abstract

Pointing is one of the first conventional means of communication and infants have various motives for engaging in it such as imperative, declarative, or informative. Little is known about the developmental paths of producing and understanding these different motives. In our longitudinal study (*N* = 58) during the second year of life, we experimentally elicited infants’ pointing production and comprehension in various settings and under pragmatically valid conditions. We followed two steps in our analyses and assessed the occurrence of canonical index-finger pointing for different motives and the engagement in an ongoing interaction in pursuit of a joint goal revealed by frequency and multimodal utterances. For understanding the developmental paths, we compared two groups: typically developing infants (TD) and infants who have been assessed as having delayed language development (LD). Results showed that the developmental paths differed according to the various motives. When comparing the two groups, for all motives, LD infants produced index-finger pointing 2 months later than TD infants. For the engagement, although the pattern was less consistent across settings, the frequency of pointing was comparable in both groups, but infants with LD used less canonical forms of pointing and made fewer multimodal contributions than TD children.

## 1. Introduction

A pointing gesture is among the first conventional communicative means to emerge early in a child’s development and often before the use of first words. Indeed, first pointing gestures can be observed between the ages of 9 and 12 months [1,2,3], even when infants are growing up in different cultures [4]. Whereas the literature reports some first forms such as just finger (but not arm) extension or whole-hand points [5,6,7], the canonical pointing gesture [6,7,8] “is considered to be an extended index finger with the remaining fingers curled and the arm extended” [9] (p. 112). This canonical form reflects a more advanced form of communication compared to noncanonical forms because by 12 months of age it is more frequent, more often accompanied by vocalizations, and correlates strongly with the comprehension of pointing [7].

Regarding the relation between the emergence of pointing gestures and language development, a longitudinal study by Lüke et al. [10] has revealed that, compared to infants with typical language development (TD), young infants who have been identified as language delayed (LD) at 24 months of age use *fewer* pointing gestures at the beginning of their second year of life, but *more* pointing gestures toward the end of their second year. Infants, who do not produce pointing gestures with their index finger at 12 months, are at a higher risk for language delay at 24 months performing poorer in language than TD children at least until the age of six years [10,11,12,13].

In addition to the form and frequency of pointing, infants’ vocalizations that accompany their pointing (multimodal utterances) have been shown to relate to children’s linguistic skills [14,15,16]. Murillo and Belinchón [15], for example, reported that the total number of pointing gestures in 12-month-olds observed in structured play situations related to the number of words the infants used in similar situations at 15 months. However, even stronger associations were found for pointing used in a combination with verbal utterances (vocalizations and words) and gaze. Similar results were reported by Igualada and colleagues [14] for 12-month-old infants observed in different experimental conditions and by Wu and Gros-Louis [16] who studied the use of pointing accompanied by vocalizations in 12-month-old infants within naturalistic mother–infant interactions. The authors concluded that multimodal utterances are linguistically more complex, see also [17]. In a similar vein, Liszkowski and Tomasello [7] found that infants are more likely to vocalize when pointing with the index finger than the whole hand.

From a pragmatic perspective, infants express a communicative goal through multimodal utterances “in a more complete fashion” [18] (p. 218). Multimodal utterances serve to realize various communicative goals, which is reflected by the fact that infants point within various situations. Bates et al. [19] first established that infants point within various situations for different reasons (declarative and imperative). Based on an extensive empirical program over the past decades, Liszkowski and colleagues have suggested that infants’ pointing, both in production and comprehension entails several intertwined layers of cooperative goals to (i) address each other, (ii) refer each other to entities and events, and (iii) do this to make one do/feel/know something relevant to the ongoing social interaction [20] (for an overview). Similarly, Rohlfing and colleagues [21] proposed that when using pointing, infants demonstrate not only shared attention in a moment of reference but also the pragmatic ability to use relevant communicative means to pursue an underlying interactive goal. As Franco and Butterworth [6] (p. 333) put it, pointing “appears also related to the planning and interco-ordination of sequences of actions”. This coordination becomes visible in microbehaviors that have been observed to accompany pointing such as “visual checking” [6]. This coordination also becomes visible in larger units of behaviors consisting of a sequence targeting an interactive goal. A goal can be hidden and requires the observer to relate the gesture not only to the pointed entity but also “to the situation” [18] (p. 217).

With respect to joint goals as larger units of behavior, research has grouped pointing gestures into different performative acts [1] or motives [22]. Imperative pointing was first described as using the partner as a tool to obtain an object [1]. Subsequent research found that infants point imperatively even in situations, in which they could easily access the object themselves [23] suggesting that it is used as a means within joint collaborative interaction. Declarative pointing was first described as using an object as a means to gain adult attention [1] but was subsequently discussed as having a dual function, being either expressive, in that infants share an attitude, emotional feeling, or interest with a communication partner [23], or being informative and providing the partner with needed information [24]. A further pointing motive is discussed as having an interrogative function, characterized by infants pointing for the purpose of obtaining (verbal) information [22,25,26].

In sum, pointing thus extends clearly beyond joint attention and reflects a communicative means for contributing to the accomplishment of various joint goals. In this extended view, a child does not only point but also engages (before or after the point) with an interaction partner to work toward a goal in action sequences [27,28]. A goal can be achieved in pursuit of various purposes.

Some studies have addressed the extent to which engaging for different purposes is related to the development of communication skills. A meta-analysis by Colonnesi et al. [29] investigated the relation between the different pointing motives and infants’ paths in language development. It revealed that infants who pointed in a canonical way and were driven by their declarative motive demonstrated better language skills at a later age (see also [30,31]). However, the predictive value of imperative pointing could not be evaluated, because only three out of 25 integrated studies had analyzed imperative pointing, and the results were inconclusive (e.g., [32]). Thus, both hand form and motives of pointing seem to reflect different communicative complexities and play differential roles in language and communication development. However, the studies considered so far have not compared infants’ ability to engage in interaction for different motives.

Recently, Adamson and colleagues [33] found that engagement in interaction related more strongly to later expressive vocabulary than joint attention skills in children with developmental delays (including children with developmental language disorder, DLD) and children with autism spectrum disorders (ASD). Children with language delay (LD) have a much higher risk for DLD than TD children [34]. Children with DLD have language deficits with a significant impact on social interactions and educational success [35]. For older children with a developmental language disorder (DLD), research reveals higher social withdrawal than in TD children. More specifically, parents of preschoolers with DLD rated their child’s level of cooperation lower than parents of TD children [36]. However, they were observed to gesture more within interactions with their parents [37]. Following this line, the question is whether infants with LD join an interaction less readily. However, this has hardly been investigated. Addressing interactive engagement, yet focusing on children with ASD, Tomasello and Camaioni [38] observed less pointing for declarative motives compared to TD children. In comparison to the group of children with ASD, those with DLD were reported to respond in a typical manner to joint attention interaction and to have more communicative and advanced gestural behavior [39]. Engagement in social interaction, thus, might be a differentiating factor between children with DLD and ASD. Hence, more research is needed to clarify the link between engagement in communication and delayed language development.

Therefore, in the present study, we aimed to shed light on the developmental paths of pointing motives in infants by asking the following research questions: First, what are the ages of occurrence of pointing for different motives and of comprehending them? Second, while contextualizing pointing in a sequence of actions leading to different joint goals [28], we also asked: What forms does infants’ multimodal engagement take when pursuing different motives? Crucial to the investigation of the two questions, we included a clinical perspective by comparing pointing performance in two groups: infants with versus without LD. From the literature, we developed two possible predictions for the second question: If communicative development includes infants’ understanding of the joint goal and infants with LD have deficits in this respect, we should see a delay in this communicative behavior in the given group of infants in the form of responding less to the experimenter’s interactional bids, especially for the declarative motives. Another possibility is that infants with LD will respond comparably often to the experimenter’s interactional bids but in a less advanced way by, for example, relying longer on whole-hand pointing and less on accompanying vocalizations.

## 2. Materials and Methods

### 2.1. Participants

Sixty-three infants participated in this longitudinal study. Data from five infants were excluded for the following reasons: no participation after the age of 2;6 (2 infants), chronic otitis media with several effusions (1), and increasing exposure to additional languages throughout the course of the longitudinal study (2). The final sample consisted of 58 infants (30 boys, 28 girls) with a mean age of 12 months and 7 days (*SD* = 12.3 days) at the beginning of the study. Because two infants first started the longitudinal study at 14 months of age, the sample size at 12 months of age was *n* = 56. Recruitment was designed to increase the involvement of infants with language delays in the sample. Families were informed about the study via their pediatricians during their regular medical check-ups when infants were between 10 and 12 months old. The medical staff were encouraged to especially invite families with a sibling or a parent who had a history of language disorder to the study in order to increase the number of infants with a higher risk of LD [34]. This effort resulted in 14 of the 58 infants being identified as LD at 24 months of age (see Section 2.3). However, we should highlight that because of the longitudinal design, infants that were identified as late talkers [40], i.e., infants with language delay (LD) who are defined as having productive vocabularies below 50 words at 24 months of age and as not producing two-word utterances at that age (e.g., [40,41]), clearly differ from older infants who are diagnosed with DLD.

All participating infants were raised as monolingual German speakers. Their general development was rated by their pediatricians and assessed at the beginning of the study using a standardized test that covered cognitive, motor, language, social, and emotional development (Entwicklungstest für Kinder von 6 Monaten bis 6 Jahren, ET 6-6 (developmental test for infants aged 6 months to 6 years) [42]. According to both pediatricians and the standardized test results, all infants were developing typically.

### 2.2. Design and Procedure

This longitudinal study incorporates a total of 14 observation sessions over the course of 5 years, with experimental settings to elicit infants’ early verbal and gestural behavior at 12, 14, 16, 18, and 21 months of age; assessment of linguistic skills at 24 and 30 months of age; and further experimental settings and language assessments at 3;0, 3;6, 4;0, 4;6, 5;0, 5;6, and 6;0 years of age, cf. [10,11,13]. Here, we refer to the experimental data from 12 to 18 months of age, and the assessment of linguistic skills at 24 months.

#### 2.2.1. Elicitation of Pointing Gestures

The production and comprehension of infants’ pointing gestures were observed in different experimental situations designed to elicit different pointing motives. These experiments are based on previous studies [43,44,45,46]. We created the following experimental situations that vary in the motive they elicited. The numbers in the parentheses specify the order in which the experimental situations were carried out:

- Production of imperative pointing (setting 3),
- Comprehension of imperative pointing (setting 4),
- Production of declarative (expressive) pointing (setting 2),
- Production of informative pointing (setting 5),
- Comprehension of informative pointing (setting 1).

The comprehension of declarative pointing (using a point-following task) was assessed at 12 months of age only. This was based on previous research indicating that infants are largely able to follow a pointing gesture by 12 months of age [47]. Therefore, this situation is not included in the analyses.

In all settings, the child sat in a highchair facing a female experimenter. The experimenter sat at a table with a white cloth screen behind her (2.30 m × 2.50 m). The screen had four windows (2 left and 2 right; each 25 cm × 25 cm) and was used in setting 2.

The order of the experimental situations was fixed (see numbers in the parentheses in the list above), and four trials were performed with different objects in each situation presented in random order. The situations eliciting declarative and imperative pointing production have been reported in a previous publication [11] and are based on the procedure used by Liszkowski, Carpenter, and Tomasello [43] for declarative pointing as well as the procedure used by Camaioni et al. [45] for imperative pointing. Table 1 gives an overview of the procedures in the experimental settings.

#### 2.2.2. Coding of Gestural and Multimodal Communication

The video recordings were coded using ELAN transcription software [48]. For the comprehension tasks (settings 1 and 4), a pointing gesture was coded as being understood if the infants reacted correctly (searching at the correct location in setting 1 and handing the missing piece over or declining to hand the missing piece over by saying “no” or by head shaking in setting 4). In the production tasks (settings 2, 3, and 5), infants’ gestures were coded for different gesture types, and the semantic relation if produced together with verbal utterances, see e.g., [49]. We differentiated between two forms of pointing:

i. Index-finger pointing in the case of an arm extension and clear extension of the index finger toward an object, a person, or an event
ii. Whole-hand pointing in the case of an arm extension without clear extension of the index finger.

In cases in which, in addition to the clear extension of the index finger, other fingers were also partially extended, this was coded as whole-hand pointing. Gestures produced with an extension of the arm and repeated opening and closing of the fingers, towards an object, were coded as grabs. Because grabs and other gesture types (i.e., emblems) occurred in small numbers, they were not included in the analyses. Interrater reliability was assessed by having 10% of the data coded by a second coder. Krippendorff’s α [50] was calculated for all variables. It ranged from 0.82 to 1.00, and therefore was very good (above 0.80).

Our analyses follow two strands: First (see Section 3.1), we identified pointing as an expression occurring at a specific age by applying the dependent measure of age at first observation in our experimental settings. Second (Section 3.2), we focused on communicative engagement assessed as the mean amount of pointing reflecting how engaged infants were in situations that clearly called for their contribution.

##### Multimodal Utterances

If a gesture was produced together with a verbal utterance, this was coded with respect to the different semantic relations (reinforcing, disambiguating, adding, uttering a protoword, or vocalizing). However, for the present analysis, we did not differentiate between these forms but summed the numbers up. Furthermore, and due to our procedure of coding, multimodal communicative bids were assessed for all gestural types together in settings 2 and 3. In the setting used to elicit the production of imperative pointing (setting 3), in addition to the pointing gestures, we observed very few emblems and grab gestures that were accompanied by verbal utterances. Therefore, the analysis of the multimodal engagement includes these gesture productions.

### 2.3. Assessment of Language Delay

To assign participants in our sample to a group of infants with TD or LD, we assessed their linguistic skills at 24 months using a standardized German language test, “Sprachentwicklungstest für zweijährige Kinder (SETK-2)” (test of language acquisition for 2-year-old infants) [51]. This test consists of four subtests assessing the comprehension and production of words and sentences. In accordance with other authors [52,53], we defined a 2-year-old child as being language delayed if she or he scored 1.5 *SD* below the mean (i.e., T-score of ≤35) in at least one of the four subtests and 1 *SD* below the mean (i.e., T-score of <40) in at least one additional subtest. The recruitment strategy resulted in 24% of the infants in the sample having a primary language delay at 24 months.

## 3. Results

### 3.1. Developmental Paths of Gestures as Communicative Expression in the Context of Different Motives Elicited Experimentally in TD and LD Infants

Our first analysis addresses whether LD infants are delayed in expressing and understanding different pointing motives compared to TD infants. To this end, we compared both groups in terms of when we first observed different pointing gestures at least once and when infants showed a robust understanding of imperative and informative gestures (i.e., at least 3 of 4 trials with correct behavior).

**Figure 1:**
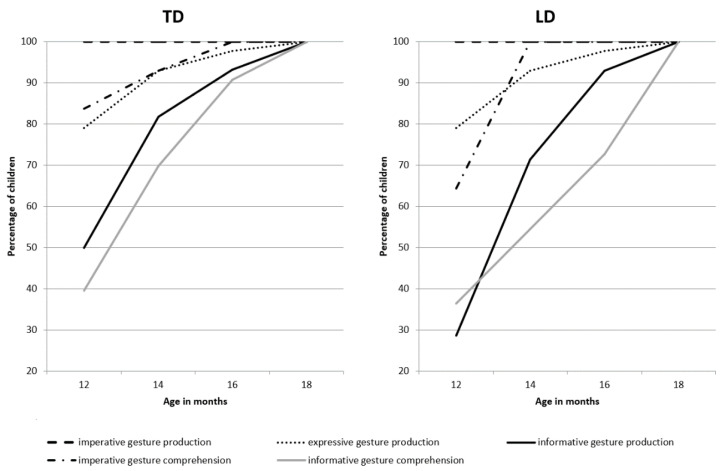
Developmental paths of gesture productions and comprehensions in the context of different motives elicited in TD infants (*n* = 44; **left**) and infants with LD (*n* = 14; **right**).

**Figure 2:**
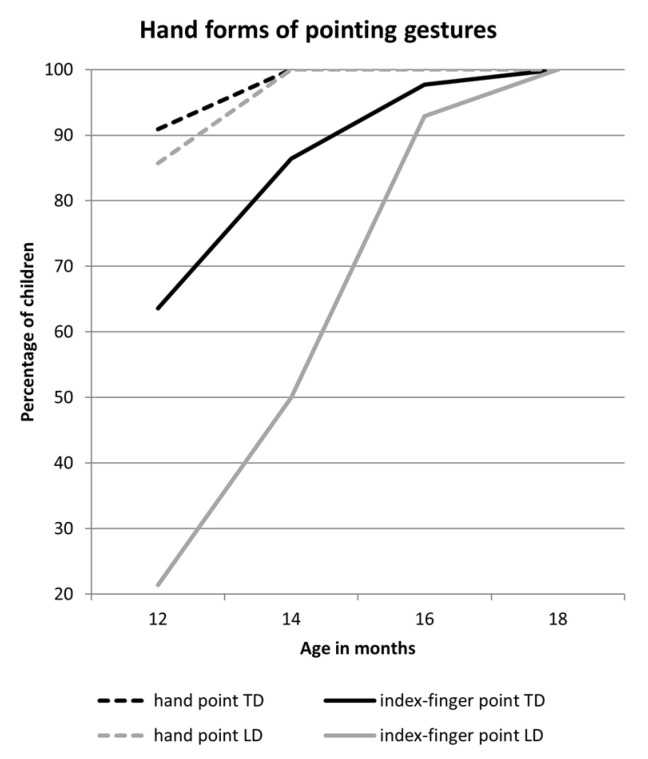
Percentage of TD infants (*n* = 44) and infants with LD (*n* = 14) using whole-hand and index-finger points in the production settings.

Figure 1 and Figure 2 show the developmental paths of pointing gestures for each group of infants separately—or more specifically, what percentage of infants in each group used and comprehended the pointing gestures at which age. The figures also display the production and the comprehension settings.

For the group comparisons of age at first observations, Mann–Whitney U tests were applied due to the different sample sizes. Table 2 reports the descriptives and test statistics.

The group comparisons showed that there was a significant difference in all production settings with respect to the age at first observation of at least one index-finger point. In the group of infants with LD, we observed the first production of index-finger points about 2 months later (i.e., one observation setting later) than in the group of the TD infants. However, there were no differences between infants with and without LD with respect to the first observation of comprehension of pointing gestures with different motives (Table 2).

### 3.2. Developmental Paths of Engagement in Multimodal Interaction

In the previous section, we reported the results of the first observation of the pointing gestures used and understood in our different experimental situations based on a binary coding, together with how the TD infants and infants with LD differed in this regard. In the following, we shall investigate the infants’ communicative engagement by analyzing the mean number of pointing gestures that the two groups of infants produced per trial (settings 2, 3, and 5) and the mean number of correct reactions in the comprehension settings (settings 1 and 4). This was necessary because a few trials could not be evaluated due to the fussiness of the infants or errors of the experimenters. We hypothesized that the infants with LD would respond less to the experimenter’s interactional bids, especially for the declarative motives, and rely longer on their gestural communication repertoire [10] consisting of pointing gestures performed without vocalization. Again, Mann–Whitney U tests were applied for the group comparisons due to the different sample sizes. Below, we shall present the results for the different motives (see Table 3 for the statistics):

For the production of the imperative motive, we found that, at 12 months of age, TD infants used their index-finger points significantly more often than infants with LD, whereas infants with LD used the whole-hand point more frequently per trial in this setting. Infants in both groups used different forms of pointing but were comparable in terms of engaging in this communicative situation, as shown by the group comparison of the sum of both hand forms of pointing. At 14 months of age, we found that TD infants used significantly more index-finger points compared to infants with LD. No group differences were found with respect to the comprehension of imperative pointing (setting 4) at any age.

For the declarative motive, we found group differences in the production of index-finger pointing only at 12 months of age, and not at the other observation timepoints. Taking all hand forms together, both groups engaged to a similar extent.

The group differences at 12 months of age in index-finger pointing with the imperative and declarative motive are probably due to the fact that most LD infants did not use any index-finger points at 12 months (see Section 3.1). However, of the 44 TD infants, there were also many infants who did not produce index-finger points at 12 months of age in one of the settings: 19 in the imperative setting and 26 in the declarative setting.

No significant differences between both groups were found for the number of pointing gestures produced in the informative setting at 12, 14, or 16 months of age because both groups produced very few gestures in this setting until 18 months. At 18 months, the groups differed significantly in their number of index-finger points: TD infants used significantly more index-finger points per trial than infants with LD. Again, when taking all hand forms together, we found no group differences.

For the comprehension of the informative pointing, we found a significant group difference at 16 months of age, suggesting that at this age, TD infants engage more in the comprehension of informative pointing than infants with LD—an effect that is no longer observable 2 months later.

For the multimodal communication, the combinations of gestures and words were calculated proportionally to the mean number of gestures produced per trial to control for individual differences in gesture frequencies. Group differences were found at 14 months of age in the imperative setting (*Md_TD_* = 0.14, *IQR* = 0.52; *Md_LD_* = 0, *SD* = 0.08, *U* = 199.5, *p* = 0.034, *d_Cohen_* = 0.78) and at 18 months of age in the declarative setting (*Md_TD_* = 0.47, *IQR* = 0.57; *Md_LD_* = 0, *SD* = 0.33, *U* = 158, *p* = 0.01, *d_Cohen_* = 0.11). In both cases, infants with LD produced significantly fewer gesture–speech combinations per trial compared to TD infants.

## 4. Discussion

In the current study, we investigated the developmental paths of the occurrence of pointing for different motives. Furthermore, we asked how infants engage multimodally for the different motives. We used experimental settings that established a pragmatically meaningful need for infants with and without LD. We followed two steps in our analyses by first considering the first occurrence of canonical pointing gestures in the experimental settings, and second, the frequency with which infants use pointing gestures to engage in a joint goal with and without vocalizations. The second step took a broader focus and considered a pointing gesture as engagement in an ongoing interaction following a joint goal. This was captured by the frequency of pointing and accompanying vocalizations in different settings. We then tested whether the two groups of infants differed in their engagement in social interaction.

The first analysis revealed that infants’ pointing behaviors that were elicited by the three settings differed in their age of occurrence, with pointing for the imperative motive already being in place at 12 months of age, and declarative expressive and informative motives occurring later at 14 months of age for the majority of typically developing infants.

When we compared across the two groups, we found considerable intermediate effects and significant differences in all production settings that pertain to the first observation of index-finger points: The group of LD infants produced their index-finger points 2 months later (i.e., one observation setting later) compared to the group of the TD infants. These findings extend our previous work showing that index-finger pointing at 12 months of age is predictive of a typical language development [11]. The current analysis also shows that the group of infants with LD use index-finger pointing significantly later (about 2 months) in each of the three settings.

Regarding our second analysis of pointing as engagement, we found that when all forms of pointing are taken into consideration, infants of both groups engaged in interaction to a similar extent. However, we found two important differences when looking at the interaction engagement: Infants with LD used the canonical form of pointing less at an early age, and they combine it less frequently with the vocal behavior at a later age. This is supported by the finding for the imperative motive at 12 and 14 months suggesting that infants with LD produce significantly less index-finger points but more whole-hand points. At 14 months, their multimodal contributions are significantly less frequent than those in TD infants. A similar pattern is found for the declarative setting: At 12 months of age, the LD infants produced less index-finger points than TD infants, but they engaged comparably often using index-finger or whole-hand points. Later, at 18 months of age, they produced less multimodal utterances than their TD peers.

When comparing the settings to each other, all infants point infrequently and very little in the informative setting. It is only at the age of 18 months that we observed significantly more index-finger points in TD infants than in infants with LD. Again, however, when looking at the engagement with all means (canonical or not), no differences between the groups are apparent—an effect that we found for all settings.

Thus, our findings are interesting with respect to the proposition that infants with LD might be less expressive overall and tend to withdraw from the interaction. Indeed, extroversion has been discussed as a personality factor in speakers that possibly moderates their production of gestural behavior [54] (for persons with typical linguistic skills). However, our results do not support this proposition in either of the settings. We can only report the finding that LD infants are less engaged when comprehending informative pointing at 16 months—an effect that vanishes 2 months later. Our approach to capturing infants’ engagement, however, is limited to the frequency of their pointing. An alternative would be to create engagement settings that define infants’ roles and assess more clearly their communicative contribution by scoring it. Nevertheless, our results that children with LD and TD children engage comparably often with an adult but use their communicative means differently are in line with findings by Wray et al. [37], showing that older children with DLD use more gestures in interactions with their parents than TD children to successfully engage in an interaction.

Taking the two analytical steps together, our results are consistent with what Lüke et al. [11] found at 12 months not only in the declarative and imperative experimental setting but also in a more naturalistic setting of infants interacting with their parents: TD infants point using the canonical form earlier in their development than infants with LD. Our results on index-finger points are also in line with Aureli et al. [55] and Perucchini et al. [56] who reported no differences between imperative and declarative pointing in terms of its frequency of occurrence at one age or across ages. In support of this, whereas TD infants pointed more frequently with their index finger in both settings at 12 and 14 months than infants with LD, the group differences for multimodal contributions occurred at different ages (14 months for imperatives and 18 for declaratives).

Our results extend the findings reported by Salo et al. [31]. The authors investigated infants’ pointing behavior in natural parent–child interactions. In the coding, the authors did not differentiate between index-finger and whole-hand points, and further combined pointing for informative and declarative purposes into one category. In this respect, the present study offers a differentiation as we considered various hand forms and various motives. Further, we compared the two groups rather than related the motive-related performance with children’s later language development. In addition to Salo et al.’s findings that pointing at 12 months for the declarative motive predicts receptive and productive vocabulary at 24 months, our analysis revealed effects for all motives studied that are, however, limited to the index-finger form of pointing. When considering the frequency of all pointing forms together, no differences in engagement in children with LD compared to TD children could be found.

With respect to multimodal communicative behavior, Cochet and Vauclair [57] found an increase in pointing–vocal coupling for the declarative motive, but they studied older infants aged 15 to 30 months. We, however, found that in contrast to infants with LD, TD infants were more often coupling pointing behavior with vocalizations early in their development for imperative purposes; and 2 months later in their development, for declarative purposes. Based on our results, we propose that a pointing–vocal coupling occurs when a type of a pointing gesture is used steadily, as might have been the case for declarative pointing in Cochet and Vauclair [57]. Importantly, we found that the coupling does not start in general for all motives because they pertain to different plans and goals of interaction. Instead, pointing–vocal coupling seems to be related to infants’ mastery of pointing that is specific to a particular motive

As pointed out in the introduction, our data are limited to a sample from a Western culture. Following Salomo and Liszkowski’s [58] findings on the emergence of pointing being mediated by the frequency of joint actions, and thus emerging in terms of socio-interactional experience, future work needs to be conducted with other cultural samples to take these cultural differences into account.

## 5. Conclusions

It is important to emphasize that infants with LD can engage in an interaction with a partner in pursuit of various joint goals and with various forms of pointing. However, they master this sequence of communicative actions with index-finger pointing—a canonical form of pointing—about 2 months later than TD infants. In addition, our results suggest that pointing–vocal coupling occurs in infants when a type of pointing gesture is mastered and used steadily. Hence, our results thus indicate that index-finger pointing and an expression of more settled pointing–vocal coupling, but not the communicative engagement in an interaction, may be used to differentiate between the group of infants with and that of infants without LD.

## Figures and Tables

**Table 1:** Overview of experimental settings used in the longitudinal study.

Order	Setting	No of Trials	Items Used	Procedure	Based on	Differences to Original Study
3	Production of imperative gestures	4	2 windup toys (clown, monkey), 2 toys playing music (car, turtle)	The highchair was moved 60 cm away from the table, so that the infant was unable to reach the items on the table. E turned on one of the toys for 10 s and played with it. Afterwards, E looked at the infant silently for 15 s, then emoted positively about the toy (“Ist das nicht schön? Gefällt dir das?” [“Isn’t that pretty? Do you like it?”]) and looked at the infants for another 15 s. After this total of 30 s, the toy was given to the infant.	Camaioni et al., 2004 [45]	4 trials instead of 8.
4	Comprehension of imperative gestures	4	2 stacking towers, 2 soft toys with removeable body parts	In each trial, E put the pieces/parts of the item on the table and handed one of them to the infant. Then, E put the parts together. When noticing that one piece was missing, E said “oh”, looked at the infant, and produced an imperative reaching gesture toward her or him. If the infant did not react within 10 s by handing the piece over, E said “Das fehlt mir” [“I’m missing that”], and waited for another 10 s. As soon as the infant handed the piece over, E took it and thanked the infant.	Camaioni et al., 2004 [45]	4 trials instead of 8. We referred to the objects with the deictic term “this” instead of the label to avoid an influence of different lexical competencies.
2	Production of declarative (expressive) gestures	4	4 hand puppets (monkey, dog, cow, tiger)	A research assistant presented the hand puppets in randomized order through one of the four windows in the white cloth screen. Each trial consisted of a present and an absent phase, each 20-s long. During the present phase, E turned to the puppet and emoted positively about it, and she alternated her gaze twice between the puppet and the infant. During the absent phase, E again alternated the gaze twice between the infant and the screen for 20 s while smiling and expressing positive emotions.	Liszkowski et al., 2007 [43]	4 windows in the white screen cloth instead of 2. Further, we did not use different conditions.
5	Production of informative gestures	4	Foam puzzle, colored pencils, and paper, 2 sorting sets with warehouse items	In this setting, E engaged in different activities (drawing a picture, sorting things, doing a puzzle). While taking the material out of a box under the table, one piece was accidentally dropped down the table on the infant’s side. During the activity, E noticed that the piece was missing and said “Huch, nanu, na sowas. Das ist ja komisch!” [“Oops, that’s strange.”] E searched for 10 s while monitoring the infant for a reaction. If the child did not point to the piece on the floor, E said: “Wo ist das denn?” [“Where is it then?”], searched for another 10 s, and, if necessary, repeated the question. E waited another 10 s. If the infant pointed to the location, E stood up, picked up the piece, and thanked the child. If the infant did not point after the total of 30 s, E searched for the piece herself and picked it up.	Liszkowski et al., 2008 [46]	No warm-up trials. No distractor objects were used.
1	Comprehension of informative gestures	2 warm-up trials, 4 test trials	5 different finger puppets that could easily be hidden in one hand; colored cloths (40 cm × 40 cm)	*Warm-up trials:* The finger puppet was presented to the infant and then hidden in two containers on the left or right side respectively (see Behne et al., 2011) that were covered by two identical colored cloths. The infant could see where the puppet was placed. E did not point to the location. The finger puppet used in the warm-up trial was not used again in the test trials. *Test trials:* Similar procedure as in warm-up, with the exceptions that (a) after the finger puppet was presented, E took the puppet in both hands, put her hands under the table where she moved the puppet into the fist of one hand, and then moved both hands (fist) under one of the cloths on each hiding location. (b) E pointed and gazed toward the hiding location for 20 s without looking at the infant. If the infant searched at the correct location, this was rated as a correct search.	Behne et al., 2012 [44]	In contrast to the original procedure, we conducted only 2 warm-up trials with every child. In the original procedure, warm-up trials where repeated until every infant searched for the toys.

*Note:* E = experimenter.

**Table 2:** Comparisons of gestural abilities in TD and LD infants according to the month (*Md*), in which the abilities were first observed.

Gestural Ability	TD (*n* = 40–44)		LD (*n* = 11–14)		*U*	*p*	*d_Cohen_*
	*Md*	*IQR*	*Md*	*IQR*			
**Production**							
Imperative hand point	12	0	12	0.5	302.5	0.890	0.03
Imperative index point	12	2.0	15	2.5	171.5	0.007	0.69
Expressive hand point	12	2.0	12	2.5	276.5	0.705	0.09
Expressive index point	14	4.0	16	4.0	150.5	0.008	0.74
Informative hand point	14	4.0	14	3.0	250.0	0.458	0.19
Informative index point	14	4.0	16	4.5	159.0	0.030	0.60
**Comprehension**							
Imperative gestures	12	0	12	2.0	250.0	0.183	0.25
Informative gestures	14	4.0	14	6.0	200.0	0.411	0.22

*Note*: *Md* = median, *IQR* = inter-quartile range.

**Table 3:** Comparisons of communicative engagement of TD and LD infants in different experimental settings and at different ages. (Significant group differences are marked with *).

Gestural Ability	TD (*n* = 40–44)		LD (*n* = 11–14)		*U*	*p*	*d_Cohen_*
	*Md*	*IQR*	*Md*	*IQR*			
**Production of imperative gestures**							
Whole-hand point at 12 months *	0.75	1.25	1.88	2.38	162.0	0.040	0.57
Index-finger point at 12 months *	0.25	1.5	0	0.19	151.5	0.016	0.63
Both pointing forms at 12 months	2.13	2.69	1.88	2.19	236.0	0.575	0.15
Whole-hand point at 14 months	1.25	2.19	0.5	2.63	250.0	0.288	0.28
Index-finger point at 14 months *	0.5	1.94	0	0.44	194.5	0.034	0.56
Both pointing forms at 14 months	2.5	2.19	1.25	2.94	221.0	0.113	0.42
Whole-hand point at 16 months	1.25	1.94	2.75	2.81	239.5	0.212	0.33
Index-finger point at 16 months	0.75	1.75	1.0	1.0	301.0	0.898	0.03
Both pointing forms at 16 months	2.88	2.44	3.88	2.94	252.0	0.308	0.27
Whole-hand point at 18 months *	1.13	1.25	2.38	1.63	196.5	0.042	0.55
Index-finger point at 18 months	1.0	2.17	0.5	1.81	254.0	0.324	0.26
Both pointing forms at 18 months	2.75	1.69	3.25	2.25	279.5	0.604	0.14
**Production of declarative (expressive) gestures**							
Whole-hand point at 12 months	0.25	0.75	0.25	0.69	261.0	0.950	0.02
Index-finger point at 12 months *	0	0.75	0	0	176.0	0.037	0.48
Both pointing forms at 12 months	0.75	1.63	0.25	0.69	189.5	0.132	0.41
Whole-hand point at 14 months	0.25	1.0	0	0.88	241.5	0.243	0.30
Index-finger point at 14 months	0.25	1.0	0	0.5	249.5	0.315	0.26
Both pointing forms at 14 months	1.0	2.17	0.5	2.38	252.5	0.366	0.24
Whole-hand point at 16 months	0.25	0.56	0.25	1.25	266.0	0.575	0.14
Index-finger point at 16 months	0.38	1.25	0.38	2.69	262.0	0.533	0.16
Both pointing forms at 16 months	0.75	2.0	1.75	2.81	227.5	0.204	0.34
Whole-hand point at 18 months	0	0.5	0.38	0.75	203.5	0.070	0.47
Index-finger point at 18 months	0.75	2.25	0.25	2.44	253.5	0.437	0.21
Both pointing forms at 18 months	1.13	2.0	1.13	3.06	282.5	0.827	0.06
**Production of informative gestures**							
Whole-hand point at 12 months	0	0.25	0	0.25	240.5	0.969	0.01
Index-finger point at 12 months	0	0.19	0	0	208.0	0.322	0.19
Both pointing forms at 12 months	0.13	0.5	0	0.5	217.0	0.568	0.14
Whole-hand point at 14 months	0.25	0.5	0	0.5	250.5	0.730	0.09
Index-finger point at 14 months	0	0.25	0	0	230.5	0.293	0.23
Both pointing forms at 14 months	0.33	0.75	0.25	0.63	253.0	0.683	0.11
Whole-hand point at 16 months	0.13	0.33	0	0.5	297.0	0.829	0.05
Index-finger point at 16 months	0.25	0.75	0.13	0.5	262.5	0.381	0.22
Both pointing forms at 16 months	0.67	0.75	0.29	1.0	255.0	0.320	0.26
Whole-hand point at 18 months	0.13	0.33	0.38	0.75	207.0	0.053	0.50
Index-finger point at 18 months *	0.67	0.75	0.25	0.56	184.0	0.021	0.62
Both pointing forms at 18 months	1.0	0.5	0.75	0.5	265.0	0.386	0.21
**Comprehension**							
Informative gestures at 12 months	0.25	0.5	0.5	0.25	204.0	0.402	0.22
Informative gestures at 14 months	0.5	0.44	0.25	0.5	229.0	0.138	0.38
Informative gestures at 16 months *	0.5	0.25	0.25	0.63	162.0	0.019	0.64
Informative gestures at 18 months	0.75	0.5	0.63	0.56	264.0	0.406	0.21
Imperative gestures at 12 months	0.75	0.46	0.88	0.69	258.5	0.909	0.03
Imperative gestures at 14 months	1.0	0.31	1.0	0.25	283.5	0.957	0.01
Imperative gestures at 16 months	1.0	0	1.0	0.27	275.5	0.431	0.16
Imperative gestures at 18 months	1.0	0	1.0	0	294.0	0.727	0.07

*Note*: *Md* = median, *IQR* = inter-quartile range.

## Data Availability

The original data presented in the study are summarized in the article (Table 2 and Table 3), further inquiries can be directed to the corresponding author. The data are not publicly available due to informed consent form not including this possibility.
